# (*R*)-(1-Ammonio­prop­yl)phospho­nate

**DOI:** 10.1107/S1600536810040304

**Published:** 2010-10-20

**Authors:** José A. Fernandes, Sérgio M. F. Vilela, Filipe A. Almeida Paz

**Affiliations:** a Department of Chemistry, University of Aveiro, CICECO, 3810-193 Aveiro, Portugal

## Abstract

The title compound, C_3_H_10_NO_3_P, crystallizes in its zwitterionic form, H_3_N^+^CH(C_2_H_5_)PO(O^−^)(OH), with the asymmetric unit being composed by two of such entities (*Z*′ = 2). The crystal packing leads to a sequence of hydro­phobic and hydro­philic layers. While the hydro­phobic layer comprises the aliphatic substituent groups, the hydro­philic one is held together by a series of strong and rather directional N^+^—H⋯O and O—H⋯O hydrogen bonds.

## Related literature

For a description of the graph-set notation for hydrogen-bonded aggregates, see: Grell *et al.* (1999 ▶). For basic stereochemistry terminology, see: Moss (1996 ▶). For the biological activity of the title compound, see: Hudson & Ismail (2001 ▶). For the crystal structure of a racemic mixture containing the title compound, see: Bashall *et al.* (2010 ▶). For previous work from our research group on the assembly of coordination polymers using phospho­nic-based mol­ecules, see: Cunha-Silva, Ananias *et al.* (2009 ▶); Cunha-Silva, Lima *et al.* (2009 ▶); Cunha-Silva *et al.* (2007 ▶); Rocha *et al.* (2009 ▶); Shi, Cunha-Silva *et al.* (2008 ▶); Shi, Trindade *et al.* (2008 ▶). For a related structure, see: Fernandes *et al.* (2010 ▶). For a description of the *TOPOS* software, see: Blatov & Proserpio (2009 ▶).
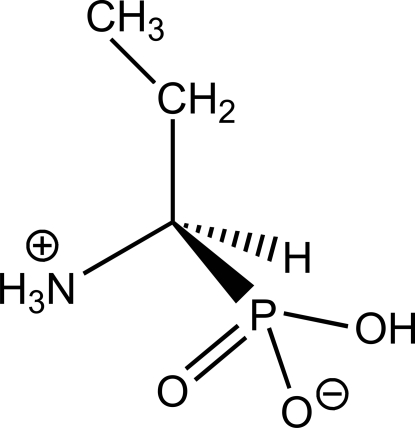


## Experimental

### 

#### Crystal data

C_3_H_10_NO_3_P
*M* *_r_* = 139.09
Monoclinic, 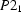

*a* = 9.3988 (13) Å
*b* = 6.2511 (8) Å
*c* = 10.8575 (15) Å
β = 105.731 (9)°
*V* = 614.02 (14) Å^3^
*Z* = 4
Mo *K*α radiation
μ = 0.37 mm^−1^
*T* = 150 K
0.16 × 0.08 × 0.02 mm

#### Data collection

Bruker X8 Kappa CCD APEXII diffractometer
Absorption correction: multi-scan (*SADABS*; Sheldrick, 1998 ▶) *T*_min_ = 0.943, *T*_max_ = 0.993
23447 measured reflections
4196 independent reflections
3465 reflections with *I* > 2σ(*I*)
*R*_int_ = 0.053

#### Refinement

*R*[*F* ^2^ > 2σ(*F* ^2^)] = 0.042
*wR*(*F* ^2^) = 0.088
*S* = 1.06
4196 reflections
171 parameters
15 restraints
H atoms treated by a mixture of independent and constrained refinement
Δρ_max_ = 0.38 e Å^−3^
Δρ_min_ = −0.55 e Å^−3^
Absolute structure: Flack (1983 ▶), 1730 Friedel pairs
Flack parameter: −0.03 (8)

### 

Data collection: *APEX2* (Bruker, 2006 ▶); cell refinement: *SAINT-Plus* (Bruker, 2005 ▶); data reduction: *SAINT-Plus*; program(s) used to solve structure: *SHELXTL* (Sheldrick, 2008 ▶); program(s) used to refine structure: *SHELXTL*; molecular graphics: *DIAMOND* (Brandenburg, 2009 ▶); software used to prepare material for publication: *SHELXTL*.

## Supplementary Material

Crystal structure: contains datablocks global, I. DOI: 10.1107/S1600536810040304/pk2268sup1.cif

Structure factors: contains datablocks I. DOI: 10.1107/S1600536810040304/pk2268Isup2.hkl

Additional supplementary materials:  crystallographic information; 3D view; checkCIF report

## Figures and Tables

**Table 1:** Selected torsion angles (°)

O3—P1—C1—C2	33.53 (18)
P1—C1—C2—C3	64.8 (2)
O6—P2—C4—C5	−77.88 (16)
P2—C4—C5—C6	170.61 (16)

**Table 2:** Hydrogen-bond geometry (Å, °)

*D*—H⋯*A*	*D*—H	H⋯*A*	*D*⋯*A*	*D*—H⋯*A*
O3—H3*D*⋯O4^i^	0.94 (1)	1.66 (1)	2.583 (2)	168 (3)
N1—H1⋯O1^ii^	0.94 (1)	1.83 (1)	2.767 (2)	178 (2)
N1—H2⋯O2^iii^	0.95 (1)	1.97 (2)	2.794 (2)	144 (2)
N1—H3⋯O4^iv^	0.95 (1)	1.91 (1)	2.843 (3)	166 (2)
O6—H6*A*⋯O1	0.94 (1)	1.65 (1)	2.589 (2)	175 (3)
N2—H4⋯O4^v^	0.95 (1)	2.05 (2)	2.914 (2)	151 (2)
N2—H5⋯O5^vi^	0.95 (1)	1.78 (1)	2.697 (2)	162 (2)
N2—H6⋯O2^vi^	0.95 (1)	1.84 (1)	2.783 (2)	172 (2)

Symmetry codes: (i) 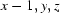; (ii) 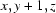; (iii) 
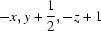
; (iv) 
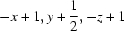
; (v) 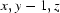; (vi) 
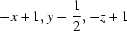
.

**Fig. 1.**
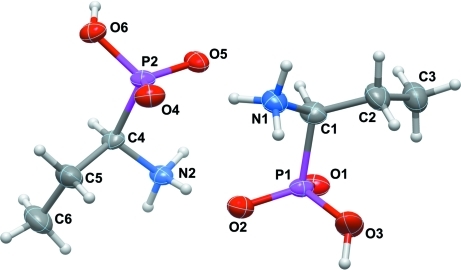
Asymmetric unit of the title compound showing the two crystallographically indenpendent molecular units. Thermal ellipsoids are drawn at the 80% probability level and the atomic labeling is provided for all non-hydrogen atoms. Hydrogen atoms are represented as small spheres with arbitrary radius.

**Fig. 2.**
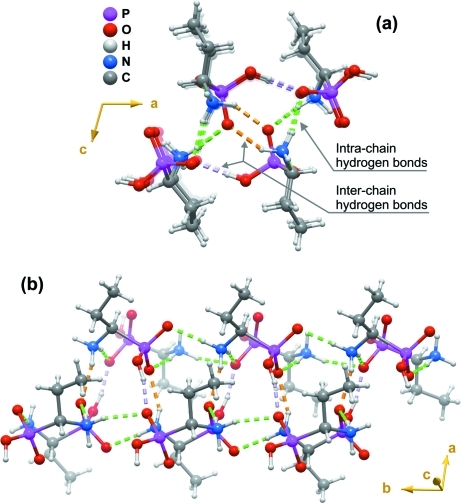
Portion of the supramolecular bilayer showing the one-dimensional zigzag chain running parallel to the [010] direction of the unit cell (graph set motif R34(10) - dashed green lines) which is interconnected by the inter-chain O—H···O (violet) and N+—H···O (orange) hydrogen bonds. For hydrogen bonding geometrical details see Table 1.

**Fig. 3.**
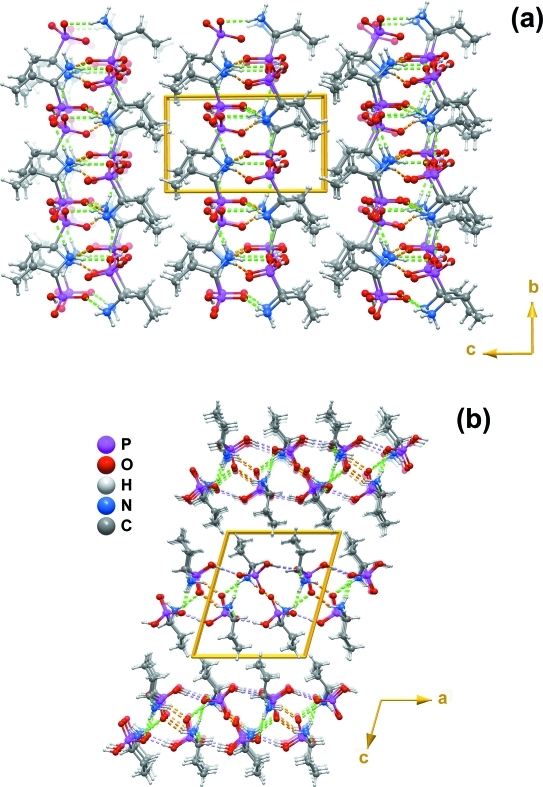
Crystal packing of the title compound viewed in perspective along the (a) [100] and (b) [001] directions of the unit cell. Hydrogen bonds are represented as dashed green (intra-chain N+—H···O), violet (inter-chain O—H···O) or orange (inter-chain N+—H···O) lines.

## supplementary crystallographic information

### Comment

The racemic mixture of the title compound is known as Ampropylfos and has been the object of several studies for its use as a pesticide (Hudson & Ismail, 2001), and its crystal structure has been very recently reported (Bashall *et al.*, 2010). Following our interest in the use of phosphonic acid molecules (Fernandes *et al.*, 2010; Cunha-Silva, Ananias *et al.*, 2009; Cunha-Silva, Lima *et al.*, 2009; Rocha *et al.*, 2009; Shi, Cunha-Silva *et al.*, 2008; Shi, Trindade *et al.*, 2008; Cunha-Silva *et al.*, 2007) here we wish to describe the crystal structure of the title compound (see Scheme).

The asymmetric unit is composed by two entities of the title compound in their zwitterionic form, in which the acidic phosphonate moiety donates one proton to the amino group (Figure 1). The geometrical conformations of the two molecules are considerably different: in one unit the two torsion angles O3—P1—C1—C2 and P1—C1—C2—C3 are both +synclinal and in the other the analogous torsion angles (O6—P2—C4—C5 and P2—C4—C5—C6) are -synclinal and antiperiplanar, respectively (see Table 1; Moss, 1996). The two crystallographically unique molecules are organized in the crystal structure into a supramolecular bilayer (in the *ab* plane) having the hydrophilic portion in the interior (composed by the amino, methyne and phosphonate moieties) and the hydrophobic in the outer position (formed by the pendant —CH_2_CH_3_ groups) (Figures 2 and 3).

Inside the hydrophilic section, individual functional groups are disposed in a zigzag fashion along the [010] direction of the unit cell, leading to the formation of a supramolecular chain held together by a combination of four N^+^—H···O hydrogen bridges (green dashed bonds in Figure 2; Table 2) - graph set motif *R^3^_4_(10)* (Grell *et al.*, 1999). Supramolecular chains are, in turn, interconnected in the *ab* plane *via* the remanant N^+^—H···O (orange dashed lines in Figure 2) and O—H···O hydrogen bonds (violet dashed lines in Figure 2).

Noteworthy, all hydrogen bonding interactions are rather strong with the internuclear D···A distances ranging from 2.583 (2) to 2.914 (2) Å. In addition, the 〈(DHA) angles range from *ca* 144 to 177°. One acceptor atom (O4) participates in a *D^1^_3_(7)* graph set motif with all 〈(DHA) greater than *ca* 150°. Other acceptors (O1 and O2) are, in turn, involved in *D^1^_2_(5)* motifs: N1—H2···O2 with 〈(DHA) of *ca* 144° and the other three interactions having angles larger than *ca* 171°. O5 is the only acceptor in a *S^1^_1_* graph set motif: N2—H5···O5 with 〈(DHA) of *ca* 162° (See Table 2).

The crystal can be better described by employing a topological approach for the description of the aforementioned hydrogen bonding interactions. Taking the geometrical centre of each molecular unit as a node, and being the hydrogen bonding interactions the connections between nodes, the structure can be simplified into a two-dimensional uninodal 7-connected single-penetrated planar (4,4)IIIb network, with total Schläfli symbol 3^6^.4^12^.5^3^ (Blatov & Proserpio, 2009).

### Experimental

The title compound was purchased from Sigma-Aldrich (Aldrich, 98%) and was used as received without purification. Suitable single crystals were grown from an aqueous solution over a period of one month.

^1^H-NMR (300.13 MHz, D_2_O) δ: 0.91 (t, 3H, *J*(^1^H-^1^H) = 7.5 Hz, CH_3_), 1.56–1.63 and 1.75–1.86 (2 m, 2H, CH_2_) and 2.96–3.05 (m, 1H, CH).

^13^C-NMR (75.47 MHz, D_2_O) δ: 13.0 (d, *J*(^13^C-^31^P) = 9.7 Hz, CH_3_), 24.7 (d, *J*(^13^C-^31^P)= 1.6 Hz, CH_2_) and 53.5 (d, *J*(^13^C-^31^P)= 143.3 Hz, CH).

^31^P-NMR (121.49 MHz, D_2_O) δ: 14.1 (dt, *J*(^31^P-^1^H)= 12.1 and 23.1 Hz).

### Refinement

Hydrogen atoms bound to carbon were included in the final structural model using a riding-motion approximation with C—H = 1.00 Å (tertiary C—H), 0.99 Å (—CH_2_) or 0.98 Å (terminal —CH_3_). The isotropic thermal displacement parameters for these atoms were fixed at 1.2 (for the two former families) or 1.5 (for the terminal methyl group) times *U*_eq_ of the respective parent atom.

Hydrogen atoms associated with the protonated —NH_3_^+^ group or the pendant —OH moiety were directly located in difference Fourier maps and were included in the final structural model with the distances restrained to 0.95 (1) Å and *U*_iso_*=1.5×U*_eq_ of the respective parent atom. The H···H distances of the —NH_3_^+^ terminal group have been further restrained to 1.55 (1) Å in order to ensure a chemically reasonable geometry.

A total of 1730 estimated Friedel pairs have not been merged and were used as independent data for the structure refinement. The Flack parameter (Flack, 1983) converged to -0.03 (8), ultimately assuring a valid absolute structure determination from the single-crystal data set.

### Crystal data

**Table:**

C_3_H_10_NO_3_P	*F*(000) = 296
*M**_r_* = 139.09	*D*_x_ = 1.505 Mg m^−^^3^
Monoclinic, *P*2_1_	Mo *K*α radiation, λ = 0.71073 Å
Hall symbol: P 2yb	Cell parameters from 6291 reflections
*a* = 9.3988 (13) Å	θ = 2.3–32.1°
*b* = 6.2511 (8) Å	µ = 0.37 mm^−^^1^
*c* = 10.8575 (15) Å	*T* = 150 K
β = 105.731 (9)°	Plate, colourless
*V* = 614.02 (14) Å^3^	0.16 × 0.08 × 0.02 mm
*Z* = 4	

### Data collection

**Table:**

Bruker X8 Kappa CCD APEXII diffractometer	4196 independent reflections
Radiation source: fine-focus sealed tube	3465 reflections with *I* > 2σ(*I*)
graphite	*R*_int_ = 0.053
ω and φ scans	θ_max_ = 33.1°, θ_min_ = 3.8°
Absorption correction: multi-scan (*SADABS*; Sheldrick, 1998)	*h* = −14→14
*T*_min_ = 0.943, *T*_max_ = 0.993	*k* = −7→9
23447 measured reflections	*l* = −16→16

### Refinement

**Table:**

Refinement on *F*^2^	Secondary atom site location: difference Fourier map
Least-squares matrix: full	Hydrogen site location: inferred from neighbouring sites
*R*[*F*^2^ > 2σ(*F*^2^)] = 0.042	H atoms treated by a mixture of independent and constrained refinement
*wR*(*F*^2^) = 0.088	*w* = 1/[σ^2^(*F*_o_^2^) + (0.0396*P*)^2^ + 0.1217*P*] where *P* = (*F*_o_^2^ + 2*F*_c_^2^)/3
*S* = 1.06	(Δ/σ)_max_ = 0.001
4196 reflections	Δρ_max_ = 0.38 e Å^−^^3^
171 parameters	Δρ_min_ = −0.55 e Å^−^^3^
15 restraints	Absolute structure: Flack (1983), 1730 Friedel pairs
Primary atom site location: structure-invariant direct methods	Flack parameter: −0.03 (8)

### Special details

**Table:**

Geometry. All e.s.d.'s (except the e.s.d. in the dihedral angle between two l.s. planes) are estimated using the full covariance matrix. The cell e.s.d.'s are taken into account individually in the estimation of e.s.d.'s in distances, angles and torsion angles; correlations between e.s.d.'s in cell parameters are only used when they are defined by crystal symmetry. An approximate (isotropic) treatment of cell e.s.d.'s is used for estimating e.s.d.'s involving l.s. planes.
Refinement. Refinement of *F*^2^ against ALL reflections. The weighted *R*-factor *wR* and goodness of fit *S* are based on *F*^2^, conventional *R*-factors *R* are based on *F*, with *F* set to zero for negative *F*^2^. The threshold expression of *F*^2^ > σ(*F*^2^) is used only for calculating *R*-factors(gt) *etc*. and is not relevant to the choice of reflections for refinement. *R*-factors based on *F*^2^ are statistically about twice as large as those based on *F*, and *R*- factors based on ALL data will be even larger.

### Fractional atomic coordinates and isotropic or equivalent isotropic displacement parameters (Å^2^)

**Table:**

	*x*	*y*	*z*	*U*_iso_*/*U*_eq_	
P1	0.17626 (5)	0.89317 (8)	0.65337 (5)	0.01280 (11)	
O1	0.29018 (16)	0.7215 (2)	0.69950 (15)	0.0197 (3)	
O2	0.10172 (14)	0.8923 (3)	0.51232 (13)	0.0182 (3)	
O3	0.06166 (16)	0.8928 (3)	0.73443 (14)	0.0272 (4)	
H3D	−0.0346 (16)	0.837 (4)	0.707 (3)	0.041*	
N1	0.1977 (2)	1.3190 (3)	0.60484 (18)	0.0159 (4)	
H1	0.231 (2)	1.455 (2)	0.6360 (19)	0.024*	
H2	0.0945 (11)	1.307 (4)	0.595 (2)	0.024*	
H3	0.217 (2)	1.294 (4)	0.5245 (13)	0.024*	
C1	0.2726 (2)	1.1499 (4)	0.69718 (19)	0.0162 (4)	
H1A	0.3742	1.1320	0.6863	0.019*	
C2	0.2910 (3)	1.2237 (4)	0.8348 (2)	0.0218 (5)	
H2A	0.3340	1.3693	0.8453	0.026*	
H2B	0.1924	1.2317	0.8509	0.026*	
C3	0.3897 (3)	1.0758 (4)	0.9338 (2)	0.0248 (5)	
H3A	0.3438	0.9341	0.9287	0.037*	
H3B	0.4021	1.1351	1.0196	0.037*	
H3C	0.4865	1.0631	0.9167	0.037*	
P2	0.64788 (6)	0.66572 (8)	0.65107 (5)	0.01273 (11)	
O4	0.78570 (16)	0.7909 (2)	0.65226 (15)	0.0177 (3)	
O5	0.54723 (16)	0.6182 (2)	0.52207 (13)	0.0180 (3)	
O6	0.57135 (16)	0.7875 (2)	0.74254 (14)	0.0169 (3)	
H6A	0.4679 (11)	0.771 (5)	0.725 (2)	0.025*	
N2	0.71494 (19)	0.2456 (3)	0.63228 (17)	0.0144 (4)	
H4	0.744 (2)	0.109 (2)	0.6697 (19)	0.022*	
H5	0.6217 (12)	0.230 (3)	0.5709 (16)	0.022*	
H6	0.7853 (16)	0.289 (3)	0.5885 (18)	0.022*	
C4	0.6989 (2)	0.4063 (3)	0.72982 (18)	0.0133 (4)	
H4A	0.6149	0.3588	0.7634	0.016*	
C5	0.8369 (2)	0.4151 (4)	0.8428 (2)	0.0208 (4)	
H5A	0.8269	0.5351	0.8994	0.025*	
H5B	0.9237	0.4449	0.8103	0.025*	
C6	0.8649 (3)	0.2079 (4)	0.9217 (2)	0.0298 (6)	
H6B	0.7746	0.1666	0.9443	0.045*	
H6C	0.9448	0.2312	0.9999	0.045*	
H6D	0.8932	0.0938	0.8711	0.045*	

### Atomic displacement parameters (Å^2^)

**Table:**

	*U*^11^	*U*^22^	*U*^33^	*U*^12^	*U*^13^	*U*^23^
P1	0.0114 (2)	0.0102 (2)	0.0176 (2)	−0.0005 (2)	0.00522 (17)	−0.0016 (2)
O1	0.0169 (7)	0.0114 (8)	0.0292 (8)	0.0003 (6)	0.0034 (6)	0.0005 (6)
O2	0.0167 (6)	0.0171 (7)	0.0207 (7)	−0.0024 (7)	0.0049 (5)	−0.0027 (7)
O3	0.0165 (7)	0.0427 (10)	0.0238 (8)	−0.0090 (9)	0.0079 (6)	−0.0080 (9)
N1	0.0193 (9)	0.0098 (8)	0.0178 (9)	0.0005 (7)	0.0035 (7)	−0.0007 (7)
C1	0.0172 (9)	0.0099 (9)	0.0200 (10)	0.0010 (8)	0.0025 (7)	0.0007 (8)
C2	0.0295 (12)	0.0170 (11)	0.0175 (10)	−0.0027 (9)	0.0040 (9)	−0.0034 (8)
C3	0.0323 (13)	0.0226 (12)	0.0177 (11)	−0.0008 (10)	0.0037 (9)	−0.0007 (9)
P2	0.0117 (2)	0.0087 (2)	0.0174 (2)	0.0009 (2)	0.00322 (18)	0.0000 (2)
O4	0.0141 (7)	0.0133 (7)	0.0261 (8)	−0.0006 (6)	0.0063 (6)	−0.0003 (6)
O5	0.0214 (8)	0.0124 (8)	0.0173 (7)	0.0038 (6)	0.0002 (6)	−0.0011 (6)
O6	0.0162 (7)	0.0130 (7)	0.0212 (8)	0.0013 (6)	0.0046 (6)	−0.0041 (6)
N2	0.0155 (8)	0.0094 (8)	0.0163 (9)	0.0010 (7)	0.0006 (7)	−0.0016 (7)
C4	0.0142 (8)	0.0090 (9)	0.0164 (8)	0.0006 (8)	0.0034 (7)	−0.0028 (8)
C5	0.0195 (10)	0.0186 (12)	0.0203 (10)	0.0030 (9)	−0.0014 (8)	−0.0004 (10)
C6	0.0359 (13)	0.0264 (14)	0.0217 (11)	0.0088 (10)	−0.0015 (10)	0.0037 (9)

### Geometric parameters (Å, °)

**Table:**

P1—O2	1.5014 (15)	P2—O5	1.4921 (15)
P1—O1	1.5029 (15)	P2—O4	1.5105 (15)
P1—O3	1.5649 (15)	P2—O6	1.5718 (16)
P1—C1	1.841 (2)	P2—C4	1.836 (2)
O3—H3D	0.939 (10)	O6—H6A	0.944 (10)
N1—C1	1.495 (3)	N2—C4	1.496 (3)
N1—H1	0.939 (9)	N2—H4	0.952 (9)
N1—H2	0.950 (9)	N2—H5	0.951 (9)
N1—H3	0.950 (9)	N2—H6	0.953 (9)
C1—C2	1.528 (3)	C4—C5	1.525 (3)
C1—H1A	1.0000	C4—H4A	1.0000
C2—C3	1.527 (3)	C5—C6	1.536 (3)
C2—H2A	0.9900	C5—H5A	0.9900
C2—H2B	0.9900	C5—H5B	0.9900
C3—H3A	0.9800	C6—H6B	0.9800
C3—H3B	0.9800	C6—H6C	0.9800
C3—H3C	0.9800	C6—H6D	0.9800
			
O2—P1—O1	115.51 (9)	O5—P2—O4	115.80 (9)
O2—P1—O3	111.82 (8)	O5—P2—O6	114.03 (8)
O1—P1—O3	110.39 (10)	O4—P2—O6	106.37 (9)
O2—P1—C1	109.12 (9)	O5—P2—C4	106.28 (8)
O1—P1—C1	106.29 (9)	O4—P2—C4	109.81 (9)
O3—P1—C1	102.78 (10)	O6—P2—C4	103.89 (8)
P1—O3—H3D	124.9 (18)	P2—O6—H6A	116.1 (16)
C1—N1—H1	110.3 (14)	C4—N2—H4	111.8 (14)
C1—N1—H2	107.5 (15)	C4—N2—H5	108.2 (14)
H1—N1—H2	110.3 (12)	H4—N2—H5	108.1 (12)
C1—N1—H3	109.2 (14)	C4—N2—H6	112.1 (14)
H1—N1—H3	110.3 (12)	H4—N2—H6	108.3 (12)
H2—N1—H3	109.2 (12)	H5—N2—H6	108.1 (11)
N1—C1—C2	110.49 (17)	N2—C4—C5	111.50 (17)
N1—C1—P1	109.52 (13)	N2—C4—P2	109.09 (13)
C2—C1—P1	115.69 (16)	C5—C4—P2	113.58 (15)
N1—C1—H1A	106.9	N2—C4—H4A	107.5
C2—C1—H1A	106.9	C5—C4—H4A	107.5
P1—C1—H1A	106.9	P2—C4—H4A	107.5
C3—C2—C1	113.07 (19)	C4—C5—C6	113.4 (2)
C3—C2—H2A	109.0	C4—C5—H5A	108.9
C1—C2—H2A	109.0	C6—C5—H5A	108.9
C3—C2—H2B	109.0	C4—C5—H5B	108.9
C1—C2—H2B	109.0	C6—C5—H5B	108.9
H2A—C2—H2B	107.8	H5A—C5—H5B	107.7
C2—C3—H3A	109.5	C5—C6—H6B	109.5
C2—C3—H3B	109.5	C5—C6—H6C	109.5
H3A—C3—H3B	109.5	H6B—C6—H6C	109.5
C2—C3—H3C	109.5	C5—C6—H6D	109.5
H3A—C3—H3C	109.5	H6B—C6—H6D	109.5
H3B—C3—H3C	109.5	H6C—C6—H6D	109.5
			
O2—P1—C1—N1	26.71 (17)	O5—P2—C4—N2	36.46 (15)
O1—P1—C1—N1	151.89 (14)	O4—P2—C4—N2	−89.49 (14)
O3—P1—C1—N1	−92.10 (15)	O6—P2—C4—N2	157.07 (12)
O2—P1—C1—C2	152.35 (15)	O5—P2—C4—C5	161.50 (14)
O1—P1—C1—C2	−82.47 (17)	O4—P2—C4—C5	35.56 (17)
O3—P1—C1—C2	33.53 (18)	O6—P2—C4—C5	−77.88 (16)
N1—C1—C2—C3	−170.11 (19)	N2—C4—C5—C6	−65.7 (2)
P1—C1—C2—C3	64.8 (2)	P2—C4—C5—C6	170.61 (16)

### Hydrogen-bond geometry (Å, °)

**Table:**

*D*—H···*A*	*D*—H	H···*A*	*D*···*A*	*D*—H···*A*
O3—H3D···O4^i^	0.94 (1)	1.66 (1)	2.583 (2)	168 (3)
N1—H1···O1^ii^	0.94 (1)	1.83 (1)	2.767 (2)	178 (2)
N1—H2···O2^iii^	0.95 (1)	1.97 (2)	2.794 (2)	144 (2)
N1—H3···O4^iv^	0.95 (1)	1.91 (1)	2.843 (3)	166 (2)
O6—H6A···O1	0.94 (1)	1.65 (1)	2.589 (2)	175 (3)
N2—H4···O4^v^	0.95 (1)	2.05 (2)	2.914 (2)	151 (2)
N2—H5···O5^vi^	0.95 (1)	1.78 (1)	2.697 (2)	162 (2)
N2—H6···O2^vi^	0.95 (1)	1.84 (1)	2.783 (2)	172 (2)

Symmetry codes: (i) *x*−1, *y*, *z*; (ii) *x*, *y*+1, *z*; (iii) −*x*, *y*+1/2, −*z*+1; (iv) −*x*+1, *y*+1/2, −*z*+1; (v) *x*, *y*−1, *z*; (vi) −*x*+1, *y*−1/2, −*z*+1.
